# A computational method to facilitate structure-activity relationship transfer

**DOI:** 10.1186/1758-2946-4-S1-O3

**Published:** 2012-05-01

**Authors:** Anne Mai Wassermann, Jürgen Bajorath

**Affiliations:** 1 Department of Life Science Informatics, B-IT, LIMES Program Unit Chemical Biology and Medicinal Chemistry, Rheinische Friedrich-Wilhelms-Universität Bonn, Bonn, 53113, Germany

## 

The transfer of SAR information from one chemical series to another is a challenging task in medicinal chemistry. However, this process is highly relevant for lead optimization because it is not uncommon that a compound series displaying a promising SAR has other liabilities, e.g. toxic side effects. In such cases, one would ideally like to build upon prior knowledge and utilize the available SAR information for the optimization of an alternative chemotype. For this purpose, alternative molecular core structures must be identified where corresponding chemical substitutions yield comparable SAR trends (consistent with a conserved mechanism of action). To support this process, we have developed a data mining method that, for the first time, enables the identification of alternative analog series with different core structures but corresponding substitution patterns and comparable potency progression [1]. A scoring scheme is utilized to evaluate the possibility of a successful SAR transfer to another series. In addition, a graphical representation has been designed to simultaneously monitor potency changes for multiple analog series as a consequence of defined substitutions. The method is also applicable to explore other aspects of SAR transfer such as comparative learning from multiple compound series with varying degrees of chemical exploration, which often results in suggestions for the design of new analogs. Furthermore, the approach has been applied to systematically assess SAR transfer potential in public compound data [1].
